# The comparison of manual and mechanical anastomosis after total pharyngolaryngoesophagectomy

**DOI:** 10.3389/fonc.2023.1041396

**Published:** 2023-02-27

**Authors:** Kexi Wang, Xiaotian He, Duoguang Wu, Kefeng Wang, Yuquan Li, Wenjian Wang, Xueting Hu, Kai Lei, Binghua Tan, Ruihao Liang, Qian Cai, Minghui Wang

**Affiliations:** ^1^ Department of Thoracic Surgery, Sun Yat-sen Memorial Hospital, Guangzhou, China; ^2^ Guangdong Provincial Key Laboratory of Malignant Tumor Epigenetics and Gene Regulation, Sun Yat-sen Memorial Hospital, Sun Yat-sen University, Guangzhou, China; ^3^ Department of Otolaryngology Surgery, Sun Yat-sen Memorial Hospital, Guangzhou, China

**Keywords:** total pharyngolaryngoesophagectomy, anastomosis, manual, mechanical, postoperative complications

## Abstract

**Background:**

Total pharyngolaryngoesophagectomy (TPLE) is considered as a curative treatment for hypopharynx cancer and cervical esophageal carcinomas (HPCECs). Traditional pharyngo-gastric anastomosis is usually performed manually, and postoperative complications are common. The aim of this study was to introduce a new technique for mechanical anastomosis and to evaluate perioperative outcomes and prognosis.

**Methods:**

From May 1995 to Nov 2021, a series of 75 consecutive patients who received TPLE for a pathological diagnosis of HPCECs at Sun Yat-sen Memorial Hospital were evaluated. Mechanical anastomosis was performed in 28 cases and manual anastomosis was performed in 47 cases. The data from these patients were retrospectively analyzed.

**Results:**

The mean age was 57.6 years, and 20% of the patients were female. The rate of anastomotic fistula and wound infection in the mechanical group were significantly lower than that in the manual group. The operation time, intraoperative blood loss and postoperative hospital stays were significantly higher in the manual group than that in the mechanical group. The R0 resection rate and the tumor characteristics were not significantly different between groups. There was no significant difference in overall survival and disease-free survival between the two groups.

**Conclusion:**

The mechanical anastomosis technology adopted by this study was shown to be a safer and more effective procedure with similar survival comparable to that of manual anastomosis for the HPCECs patients.

## Introduction

Hypopharyngeal and cervical esophageal carcinomas (HPCECs) remain a challenging clinical problem (1). These neoplasms are relatively rare and account for approximately 5-6% of all head and neck tumors (2). Most HPCECs are usually diagnosis at locally advance stages (70-80%) for the paucity of early symptoms and exhibit a poor prognosis (3).

Given the critical location and extensive involvement of the tumor, total pharyngoesopphagectomy (TPLE) followed by digestive reconstruction have been the most popular treatment modalities in the past (4). Definitive chemoradiotherapy (dCRT) and multimodality therapy (such as neoadjuvant chemoradiotherapy followed by surgery or surgery plus adjuvant chemotherapy) have gradually become central in the therapies of HPCECs (5, 6). It is worth noting that salvage TPLE surgery is a recommended choice for residual and recurrent disease when definitive medical treatment fails (7, 8).

TPLE surgical resection is a commonly used surgical method for cervical esophageal and hypopharyngeal cancer. However, such surgery has great trauma and high perioperative risk (6), so it is urgent to improve the surgical technique and prove its safety and effectiveness. As an effective surgical tool, stapling device has been widely used in the surgical treatment of esophageal cancer, which can greatly reduce operative time and the incidence of anastomotic fistula (9). However, hand sewing is the most commonly used anastomosis method in TPLE surgery. The main reason is that after larynpharyngectomy with total esophagectomy, only the tongue root and posterior pharyngeal wall remain in the surgical field, resulting in insufficient operating space and uneven tissue thickness, which makes it impossible to imbed the head end of stapler *in situ* for effective anastomosis.

Up to now, the application of staple device in TPLE is rarely reported. Some researchers have showed that using stapler inserted orally in the anastomosis process of TPLE (10, 11). In this study, we demonstrated a new technique for directly *in situ* anastomosis (avoiding the transoral approach) by using stapling device, and retrospectively compared perioperative and survival outcomes between mechanical and manual anastomosis in a single-center.

## Patients and methods

### Patient selection

**Figure 1:**
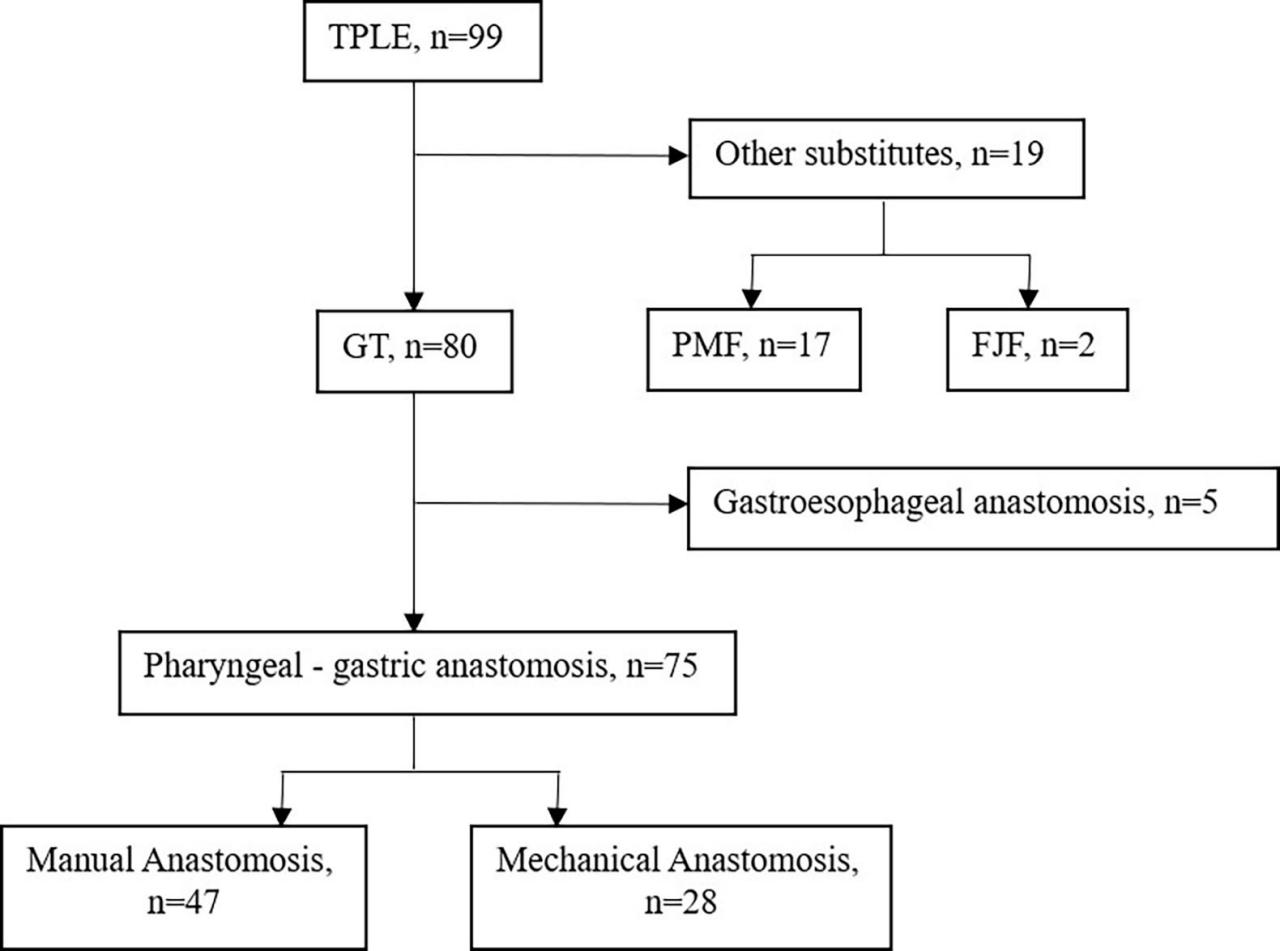
Flow chart. FJF, Free jejunal flap, GT, Gastric tube, PMF, Pectoralis major myocutaneous flap, TPLE, Total pharyngolaryngoesophagectomy.

From May 1995 to Nov 2021, a total of 99 consecutive patients with a pathological diagnosis of HPCECs and who received TPLE at Sun Yat-sen Memorial Hospital were retrospectively screened. We selected patients for surgery based on the following criteria: aged between 18 and 75 years old; diagnosed with HPCECs; clinical staged with I- IV; and with normal hematologic, hepatic, and renal function. After 17 cases of pectoralis major myocutaneous flap graft, 2 cases of free jejunal flap graft and 5 cases of gastroesophageal anastomosis were excluded, 75 cases of pharyngogastric anastomosis (47 cases of manual anastomosis, 28 cases of mechanical anastomosis) were eventually included for study (**Figure 1**).

Before surgery, a diagnosis of pathologic disease was obtained in all patients by gastroscope or direct laryngoscopy. The preoperative work-up consisted of a thorough medical history and physical examination, enhanced computed tomography scan of the neck and chest, abdominal ultrasonography, and upper gastrointestinal barium meal (as well as positron emission tomography and cranial magnetic resonance, if possible). Protocol of this study was approved by the Ethics Board of Sun Yat-sen Memorial Hospital.

### Surgical procedure

Pharyngo-laryngectomy and cervical lymphadenectomy were performed by head and neck surgeons. A standard collar incision was made in the cervical region. Complete resection of larynx, pharynx and cervical esophagus was performed with cervical lymph node dissection. Esophagectomy with mediastinal lymphadenectomy was performed *via* right thoracotomy. For patients with impaired pulmonary function, transhiatal blunt dissection was performed in the supine position. Median laparotomy was performed in parallel with the cervical procedure to construct the gastric tube.

**Figure 2:**
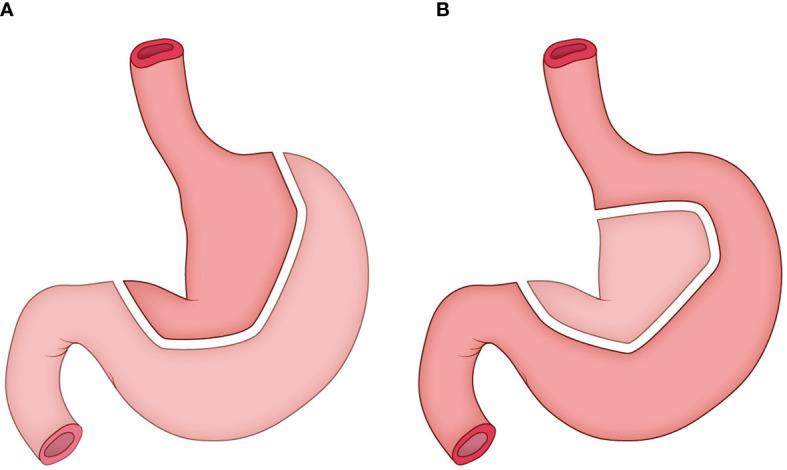
The construction of tubular stomach. **(A)** the traditional method; **(B)** the method adopted in this study.

The construction of tubular stomach starts from the lesser curvature of the stomach rather than the greater curvature, as described in our previous study (12). Briefly, the entire stomach was isolated, and a linear stapler was used to harvest both the cardia and the tissues at the lesser curvature in order to form a tube stomach with a diameter of approximately 3 cm (**Figure 2**). Then the tube stomach was inserted into the right thoracic cavity and brought up through the posterior mediastinum into the neck.

**Figure 3:**
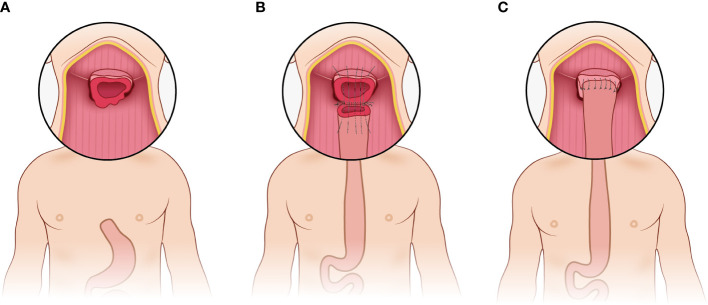
Manual anastomosis. **(A)** Exposure of the anastomotic area; **(B)** Discontinuous monolayer suturing is used for pharyngogastric anastomosis; **(C)** Completion of the manual anastomosis.

**Figure 4:**
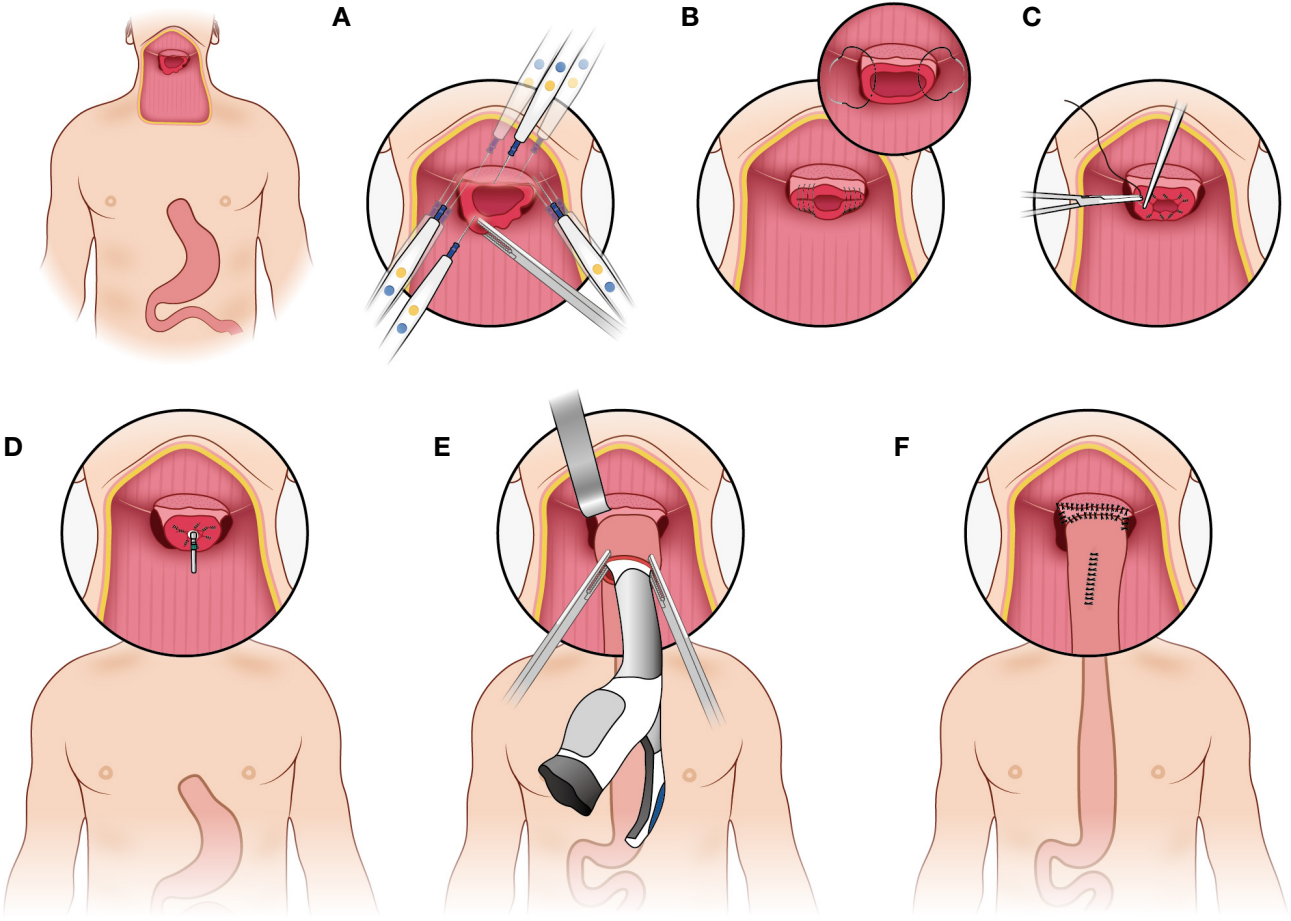
Mechanical anastomosis. **(A)** make the tongue base slightly thinned and full isolated from the posterior pharyngeal; **(B)** the anterior wall and the posterior wall were closed by intermittent suture from the laterals; **(C)** make a purse suture; **(D)** put the trocar tip of the main instrument through the middle of the opening and tighten the purse string suture; **(E)** insert the anvil into the main instrument, bring the ends together; **(F)** reinforce the anastomosis.

The pharyngogastric anastomosis is subsequently performed. In the manual group, the anastomosis was accomplished by discontinuous monolayer suturing of the muscle fibers and mucous membranes of the pharynx and stomach (**Figure 3**). For the mechanical group, the tongue base was slightly thinned and fully isolated from the posterior pharyngeal. Then the anterior wall and the posterior wall were closed by intermittent suture from the laterals until about 2cm away from the middle junction, and purse suture was made. At this point, a disposable circular stapler was introduced into the surgical field. Put the trocar tip of the main instrument through the middle of the opening and tighten the purse string suture. Insert the anvil into the main instrument, bring the ends together. After confirming again that there was no high tension and no other tissue embedded, the stapler was activated and held for several seconds. Finally, the pharyngogastric anastomosis was reinforced with simple interrupted varus suture (**Figure 4**).

### Data collection and follow-up

The baseline characteristics and outcomes of these patients were collected retrospectively. The pathological stage was defined according to the seventh edition of the American Joint Committee on Cancer TNM staging system (13). Postoperative complications were diagnosed and defined according to the Esophagectomy Complications Consensus Group (ECCG) recommendations (14). Disease-free survival (DFS) was defined as the time from surgery to disease recurrence or death. Overall survival (OS) was defined as the period from surgery to death from any disease cause or last follow-up.

Patients were followed up every 3 months in the first year and every 6 months beginning in the second year. Follow-up of patients was conducted as outpatient review and phone calls.

### Statistical analysis

Continuous variables are presented as the mean ± SD and were compared using Student’s t test or ANOVA. Categorical variables are reported as percentages and analyzed using chi-square or Fisher’s exact test. OS and DFS was assessed with Kaplan-Meier curves, compared using the log-rank test, and described as the median value at specific time points with 95% confidence intervals (CI). A 2-tailed *P*-value < 0.05 was considered statistically significant. All statistical analyses were performed using SPSS 22.0 (SPSS Inc, Chicago, IL, USA).

## Results

### Patient characteristics

This study recruited 75 patients, including 60 male and 15 female, who met inclusion criteria (**Figure 1**). Mean age was 57.6 ± 7.2 years and mean body mass index (BMI) was 20.6 ± 2.8. A total of 58 (77.3%) patients accepted surgery alone, 14 (18.7%) patients received salvage surgery after radical chemoradiotherapy failed, and 3 patients adopted neoadjuvant therapy. Esophageal blunt dissection (72.0%) and postoperative adjuvant therapy (56.0%) were performed in more than half of patients. Other variables are summarized in detail in **Table 1**. These results showed no significant differences in baseline characteristics between the two groups (28 cases in mechanical group and 47 cases in manual group, *P*>0.05).

**Table 1:** Baseline characteristics of patients undergoing TPLE.

Variables	ALL Patients (n=75)	Mechanical Anastomosis (n=28)	Manual Anastomosis (n=47)	*P* value
Age^*^	57.6 ± 7.2	58.9 ± 6.6	56.7 ± 7.6	0.252
Sex				
Male	60 (80.0)	22 (78.6)	38 (80.9)	0.811
Female	15 (20.0)	6 (21.4)	9 (19.1)	
ASA				0.496
2	37 (49.3)	16 (57.1)	21 (44.7)	
3	36 (48.0)	11 (39.3)	25 (53.2)	
4	2 (2.7)	1 (3.6)	1 (2.1)	
BMI^*^	20.6 ± 2.8	20.7 ± 2.7	20.5 ± 3.0	0.727
Weight loss>10%				0.636
Yes	14 (18.7)	6 (21.4)	8 (17.0)	
No	61 (81.3)	22 (78.6)	39 (83.0)	
Smoking				0.197
Yes	41 (54.7)	18 (64.3)	23 (48.9)	
No	34 (45.3)	10 (35.7)	24 (51.1)	
Alcohol drinking				0.883
Yes	26 (34.7)	10 (35.7)	16 (34.0)	
No	49 (65.3)	18 (64.3)	31 (66.0)	
Diabetes				1.000
Yes	3 (4.0)	1 (3.6)	2 (4.3)	
No	72 (96.0)	27 (96.4)	45 (95.7)	
Hypertension				0.726
Yes	5 (6.7)	1 (3.6)	4 (8.5)	
No	70 (93.7)	27 (96.4)	43 (91.5)	
History of malignant tumor				1.000
Yes	5 (6.7)	2 (7.1)	3 (6.4)	
No	70 (93.7)	26 (92.9)	44 (93.6)	
History of surgery				0.277
Yes	14 (18.7)	7 (25.0)	7 (14.9)	
No	61 (81.3)	21 (75.0)	40 (85.1)	
Family history of cancer				0.268
Yes	6 (8.0)	4 (14.3)	2 (4.3)	
No	69 (92.0)	24 (85.7)	45 (95.7)	
Treatment Patterns				0.723
Surgery alone	58 (77.3)	23 (82.1)	35 (74.5)	
Salvage surgery after dCRT	14 (18.7)	4 (14.3)	10 (21.3)	
Preoperative chemotherapy	2 (2.7)	1 (3.6)	1 (2.1)	
Preoperative radiotherapy	1 (1.3)	0 (0.0)	1 (2.1)	
Surgical approach				0.093
Transthoracic	21 (28.0)	11 (39.3)	10 (21.3)	
Blunt dissection	54 (72.0)	17 (60.7)	37 (78.7)	
Postoperative adjuvant therapy				0.077
Yes	33 (44.0)	16 (57.1)	17 (36.2)	
No	42 (56.0)	12 (42.9)	30 (63.8)	

*, mean ± SD; ASA, American Society of Anesthesiologists; BMI, body mass index.

### Tumor characteristics

As shown in the **Table 2**, 34 cases were cervical origin, 22 cases were cervical and hypopharyngeal origin, 6 cases were cervical and thoracic origin, 6 cases were hypopharyngeal invading to esophagus, 4 cases were thoracic and hypopharyngeal origin and 3 cases were cervicothoracic and hypopharyngeal origin in all patients. 38 patients (50.7%) had lymph node involvement and 64 patients (86.4%) had moderate differentiation. For all group, R0 resection was performed in 71 patients (94.7%), and tumor residue was found in the remaining 4 patients (5.3%). Similarly, there was no significant difference in pathological feature of tumor between the mechanical group and manual group (*P*>0.05).

**Table 2:** Tumor characteristics in the two groups.

Variables	ALL Patients (n=75)	Mechanical Anastomosis (n=28)	Manual Anastomosis (n=47)	*P* value
Location of tumor				0.125
Cervical	34 (45.3)	17 (60.7)	17 (36.2)	
Cervical and thoracic	6 (8.0)	3 (10.7)	3 (6.4)	
Cervical and hypopharyngeal	22 (29.4)	5 (17.9)	17 (36.2)	
Cervicothoracic and hypopharyngeal	3 (4.0)	1 (3.6)	2 (4.2)	
Thoracic and hypopharyngeal	4 (5.3)	0 (0.0)	4 (8.5)	
Hypopharyngeal invading to esophagus	6 (8.0)	2 (7.1)	4 (8.5)	
Lymph node invasion				0.571
Yes	38 (50.7)	13 (46.4)	25 (53.2)	
No	37 (49.3)	15 (53.6)	22 (46.8)	
None dissection of thoracic lymph nodes	54 (72.0)	17 (60.7)	37 (78.7)	
Degree of tumor differentiation				0.475
Highly differentiated	4 (5.3)	1 (3.6)	3 (6.4)	
Moderately differentiated	64 (85.4)	23 (82.1)	41 (87.2)	
Poorly differentiated	7 (9.3)	4 (14.3)	3 (6.4)	
Residual disease				0.291
R0	71 (94.7)	28 (100.0)	43 (91.5)	
R1/R2	4 (5.3)	0 (0.0)	4 (8.5)	
UICC stage of esophageal cancer				0.529
I	3 (4.0)	2 (7.1)	1 (2.1)	
II	34 (45.3)	13 (46.4)	21 (44.7)	
III	31 (41.4)	10 (35.8)	21 (44.7)	
IV	1 (1.3)	1 (3.6)	0 (0.0)	
None	6 (8.0)	2 (7.1)	4 (8.5)	
UICC stage of hypopharyngeal cancer				0.131
I	2 (2.7)	1 (3.6)	1 (2.1)	
II	2 (2.7)	0 (0.0)	2 (4.2)	
III	11 (14.6)	2 (7.1)	9 (19.2)	
IV	20 (26.7)	5 (17.9)	15 (31.9)	
None	40 (53.3)	20 (71.4)	20 (42.6)	

### Postoperative complications and surgical outcome

The rate of total postoperative complications in the mechanical group was significantly lower than in the manual group (25.0% vs 51.1%, *P*=0.027). The incidence of anastomotic fistula was 7.1% (2/28) in the mechanical group and 27.7% (13/47) in the manual group, which was significantly different (*P*=0.032). The wound infection rate was remarkably higher in the manual group than in the mechanical group (19.1% vs 0.0%, *P*=0.036). However, there was no difference in other complications, such as pneumonia, respiratory failure, postoperative bleeding and so on (*P*>0.05, as shown in **Table 3**).

**Table 3:** Postoperative complication and surgical outcome in the two groups.

Variables	ALL Patients (n=75)	Mechanical Anastomosis (n=28)	Manual Anastomosis (n=47)	*P* value
Total postoperative complications				0.027
Yes	31 (41.3)	7 (25.0)	24 (51.1)	
No	44 (58.7)	21 (75.0)	23 (48.9)	
Pneumonia				0.386
Yes	10 (13.3)	2 (7.1)	8 (17.0)	
No	65 (86.7)	26 (92.9)	39 (83.0)	
Pleural effusion				1.000
Yes	2 (2.7)	1 (3.6)	1 (2.1)	
No	73 (97.3)	27 (96.4)	46 (97.9)	
Respiratory failure				0.707
Yes	8 (10.7)	2 (7.1)	6 (12.8)	
No	67 (89.3)	26 (92.9)	41 (87.2)	
Cardio-cerebrovascular complications				0.715
Yes	2 (2.7)	0 (0.0)	2 (4.3)	
No	73 (97.3)	28 (100.0)	45 (95.7)	
Anastomotic stricture				1.000
Yes	3 (4.0)	1 (3.6)	2 (4.3)	
No	72 (96.0)	27 (96.4)	45 (95.7)	
Tracheostomal stenosis				1.000
Yes	3 (4.0)	1 (3.6)	2 (4.3)	
No	72 (96.0)	27 (96.4)	45 (95.7)	
Anastomotic fistula				0.032
Yes	15 (20.0)	2 (7.1)	13 (27.7)	
No	60 (80.0)	26 (92.9)	34 (72.3)	
Tracheal fistula				1.000
Yes	3 (4.0)	1 (3.6)	2 (4.3)	
No	72 (96.0)	27 (96.4)	45 (95.7)	
Wound infection				0.036
Yes	8 (10.7)	0 (0.0)	9 (19.1)	
No	67 (89.3)	28 (100.0)	38 (80.9)	
Postoperative bleeding				0.515
Yes	6 (8.0)	1 (3.6)	5 (10.6)	
No	69 (92.0)	27 (96.4)	42 (89.4)	
Re-operation				0.515
Yes	6 (8.0)	1 (3.6)	5 (10.6)	
No	69 (92.0)	27 (96.4)	42 (89.4)	
90-days mortality				0.291
Yes	4 (5.3)	0 (0.0)	4 (8.5)	
No	71 (94.7)	28 (100.0)	43 (91.5)	
Use of ventilator				0.994
Yes	4 (5.3)	2 (7.1)	2 (4.3)	
No	71 (94.7)	26 (92.9)	45 (95.7)	
Intraoperative blood loss (mL)	603.3 ± 500.6	389.3 ± 188.7	730.9 ± 581.1	0.001
Operation time (min)	487.9 ± 87.2	460.0 ± 81.5	504.5 ± 87.1	0.032
Postoperative hospital stays (day)	25.5 ± 13.8	21.4 ± 8.6	27.9 ± 15.8	0.046

With regard to surgical outcome, the operation time and intraoperative blood loss were significantly lower in the mechanical group than that in the manual group (460.0 ± 81.5 min vs 504.5 ± 87.1 min, *P*=0.032; 389.3 ± 188.7 mL vs 730.9 ± 581.1 mL, *P*=0.001, respectively). Similarly, the postoperative hospital stays in the mechanical group was significantly reduced than that in the manual group (21.4 ± 8.6 days vs 27.9 ± 15.8 days, *P*=0.046). For 90-days mortality, there were 4 patients in the manual group and none in the mechanical group (8.5% vs 0.0%, *P*=0.291). The causes of death include cardiac failure, pneumonia, upper gastrointestinal bleeding and uncontrolled sepsis due to anastomotic fistula. Five patients in the manual group required reoperation, compared with only one in the mechanical group (10.6% vs 3.6%, *P*=0.515). These results are shown in **Table 3**.

### Survival

**Figure 5:**
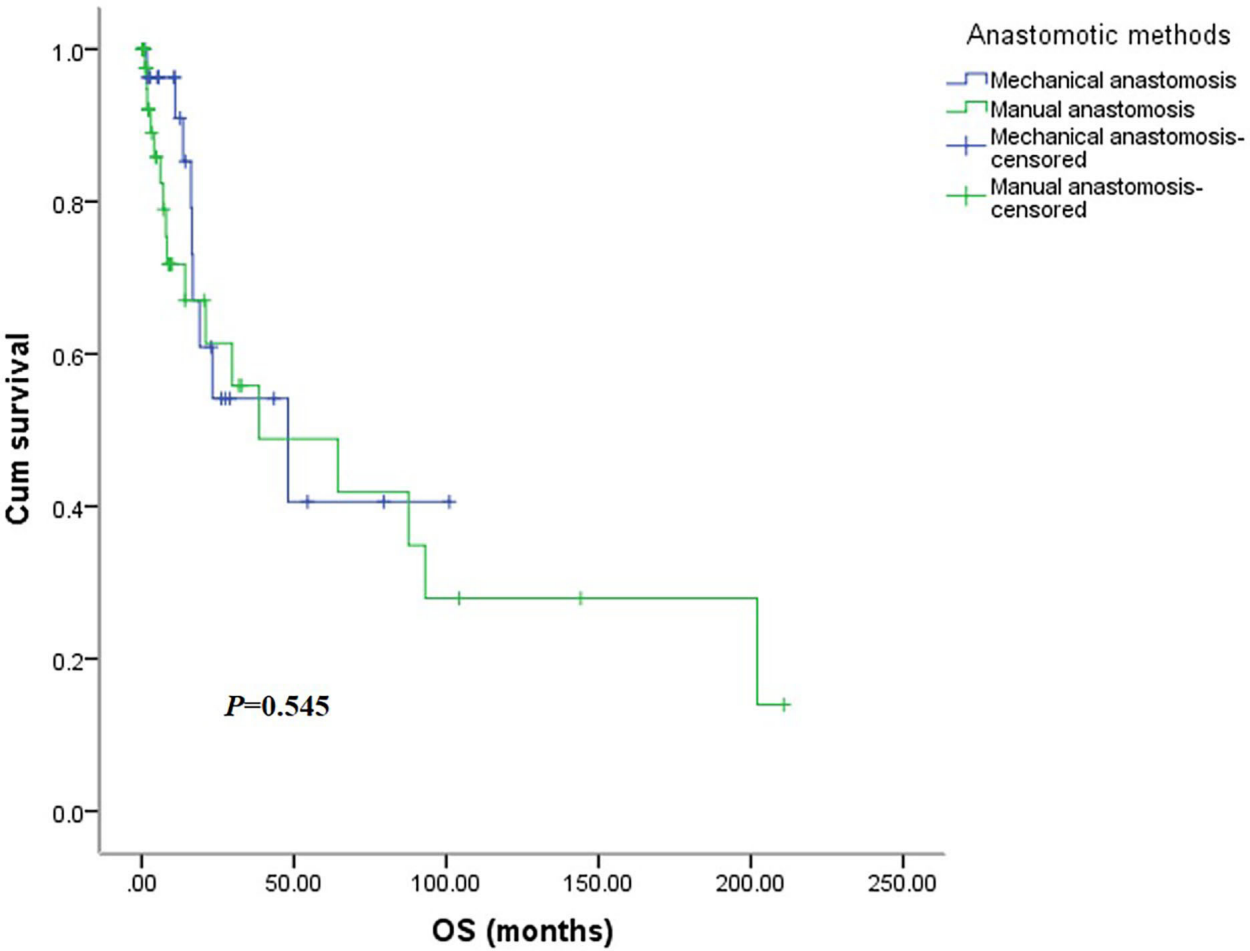
The overall survival curve of the two groups.

**Figure 6:**
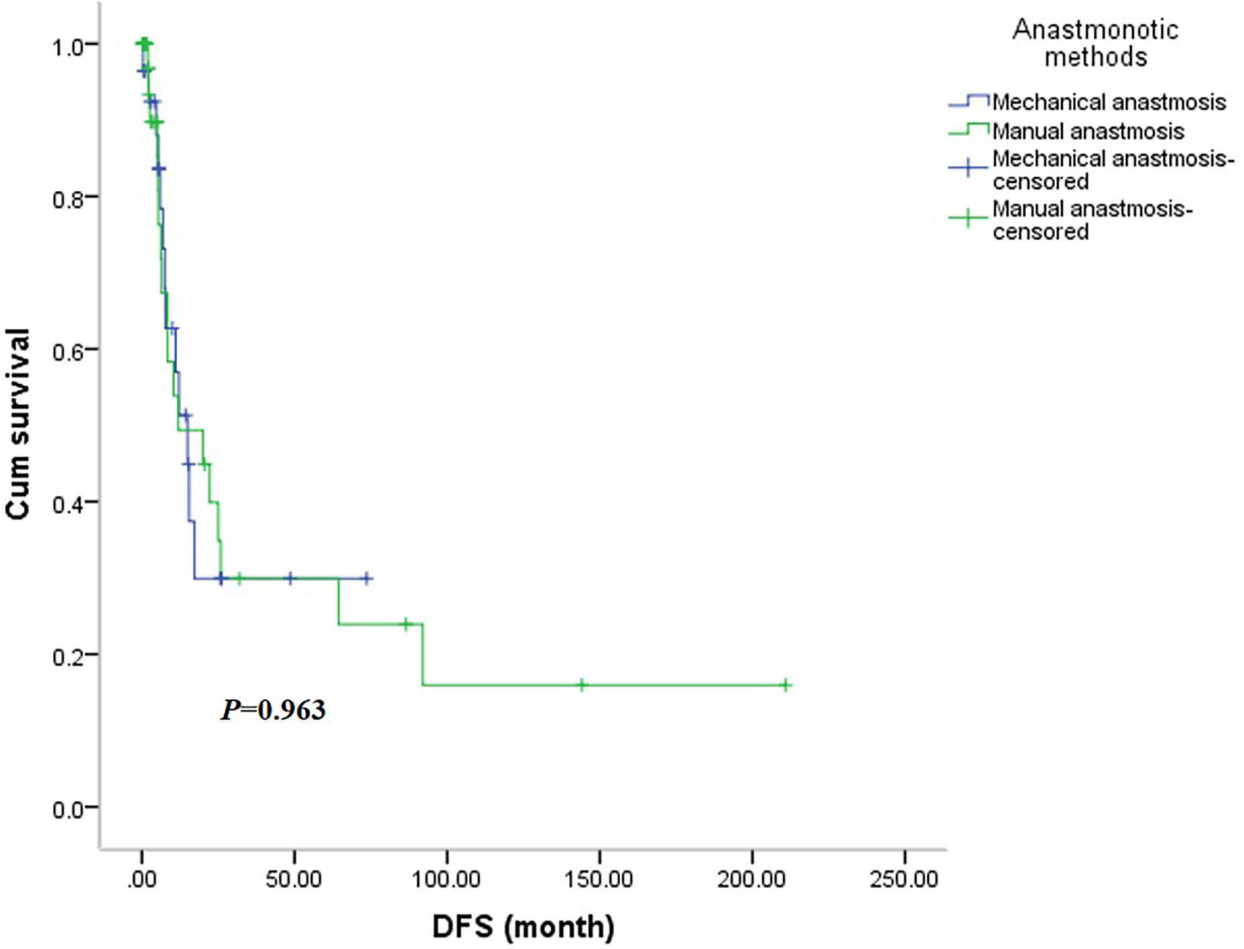
The disease-free survival curve of the two groups.

There was no significant difference in OS between the two groups (**Figure 5**), with the mechanical group surviving 48.0 months (95% CI: 6.3-89.8months) and the manual group surviving 38.5 months (95% CI: 0.0-92.7 months, *P*=0.545). Similarly, the DFS was not significantly different between the two groups (**Figure 6**), with a median survival of 15.0 months (95% CI: 7.6-22.3 months) in the mechanical group and 11.9 months in the manual group (95%CI: 0.0-29.6 months, *P*=0.963).

## Discussion

The prognosis for hypopharyngeal and cervical esophageal cancers are poor, mainly because tumors in these areas remain asymptomatic until the diseases reach an advanced stage (15). With the improvement of radiotherapy and chemotherapy technologies, locally advanced HPCECs patients can not only avoid the trauma and perioperative risk caused by surgery, but also obtain the preservation of organ function (6, 16).

However, the survival benefit of chemoradiotherapy remains unsatisfactory. The long-term survival rate of cervical esophageal cancer treated with dCRT is basically about 30% (17, 18). In addition, patients with cervical esophageal cancer who received dCRT had a high rate of local or regional treatment failure, suggesting that this treatment model has local treatment deficiency, which may be compensated by radical surgical resection to a certain extent (19). Since the result of the CROSS clinical multicenter study established the cornerstone of neoadjuvant chemoradiotherapy in esophageal cancer, the multidisciplinary treatment model has attracted increasing attention (20). Neoadjuvant therapy followed by surgery may improve the prognosis of these patients. In recent years, chemoradiotherapy has been reported as an effective treatment for advanced hypopharyngeal cancer. However, given that stage III or IV hypopharyngeal cancer often invades the cervical esophagus, and that pharygnolaryngeal and thoracic esophageal cancer frequently often occur concomitantly, surgical resection plus adjuvant therapy remains the standard of treatment (21).

TPLE is mainly indicated either for synchronous cancer of the thoracic esophagus and the head and neck or for cervical-thoracic esophageal cancer. For most patients, laryngeal preservation is not practical because their larynx and swallowing function are already impaired before treatment; In addition, most patients are diagnosed in locally advanced stages (e.g., tumor invasion of the tracheal membrane and recurrent laryngeal nerve palsy), and attempting to preserve a non-functioning larynx can also adversely affect the chances of cure. Total laryngectomy is almost always included in the surgical plan for better tumor control and postoperative recovery of swallowing function (1).

TPLE is considered to be the most complicated and most invasive surgery for surgeons due to the extremely wide resection field, long reconstructed conduit and poor blood flow of the distal end of the organ. Anastomotic fistula is the most troublesome postoperative complication in digestive tract reconstruction surgery because of its high morbidity and mortality (22). Anastomotic fistula following TPLE surgery is caused primarily by high tension and insufficient blood supply. Therefore, the application of surgical techniques becomes more important. In this study, we demonstrated a new anastomosis technique and compared it with traditional manual anastomosis for postoperative complications and survival.

Firstly, the tubular stomach was applied in this study to ensure sufficient length and adequate blood supply for pharyngo-gastric anastomosis. Our previous study showed that compared with pectoral major muscle skin flap reconstruction and whole stomach replacement, tubular gastric replacement can significantly reduce the occurrence of anastomotic fistula in patients with hypopharyngeal and cervical esophageal cancer (12).

After constructing a tubular stomach, the stomach is usually long enough to be pulled up to the neck, which may greatly reduce the tension at the anastomotic site. Then we appropriately thinned the tongue base and fully isolated the posterior pharyngeal wall, so as to provide enough space for the head end of the stapler, and then successfully performed the gastric-pharyngeal anastomosis in situ. At present, there are few reports on the comparison of anastomotic methods in TPLE for patients with HPCECs. Sallum et al. reported that the use of mechanical anastomosis (transoral approach) was effective in reducing operative time (60 min less) without additional morbidity compared with conventional manual suturing (10). Our results suggest that compared with manual anastomosis group, mechanical anastomosis group not only has significantly lower anastomotic fistula rate and wound infection rate, but also has obvious advantages of shorter operation time, less intraoperative blood loss and shorter postoperative hospital stay. Similarly, a prospective clinical study suggested that the use of stapler method reduced the incidence of leakage and shortened operating time compared with the hand-sewn method, which has been advocated as the preferred anastomotic method in esophagogastric anastomoses (9). The interpretation of this phenomenon is that mechanical anastomosis has easier operation, more uniform force, and less dependence on the stability of surgeon, while manual anastomosis has longer operation time, higher anastomotic tension, tighter suture leading to poorer blood flow, and largely dependence on the stability of surgeon. In addition, different from the stapler is introduced transorally down into the operative field for anastomosis in some studies (10, 11), we used stapler to perform anastomosis directly in situ, which is easier to operate and more time saving. In terms of survival benefit, there was no significant difference between the mechanical group and manual group in this study. In other words, we can say that the new technique of anastomosis does not affect survival or recurrence rate, but it provides safer and more effective perioperative outcomes for HPCECs patients.

In order to control the confounding factors, all surgeries were performed by the same treatment team. However, since it is a retrospective study from only single center, large-scale case studies and prospective randomized studies are still needed to further verify these results in this study.

In conclusion, by comparing the perioperative outcomes and prognosis after different methods of gastric-pharyngeal anastomosis, we concluded that the new mechanical procedure showed its advantage over the manual procedure for patients underwent TPLE in terms of less incidences of anastomotic fistula, wound infection, intraoperative blood loss, operative time and postoperative hospital stays. This reconstructive method deserves wider application and further refinement.

## Data availability statement

The raw data supporting the conclusions of this article will be made available by the authors, without undue reservation.

## Author contributions

MW and QC: Idea. KexW and XiH: Design of the study. DW and KefW: Calculation. YL, WW, and XuH: Proofread. KL, BT, and RL: Error correction. All authors contributed to the article and approved the submitted version.

## References

[1] Wang HW Chu PY Kuo KT Yang CH Chang SY Hsu WH. A reappraisal of surgical management for squamous cell carcinoma in the pharyngoesophageal junction. J Surg Oncol (2006) 93(6):468–76. doi: 10.1002/jso.20472 16615159

[2] Carvalho AL Nishimoto IN Califano JA Kowalski LP. Trends in incidence and prognosis for head and neck cancer in the united states: a site-specific analysis of the SEER database. Int J Cancer (2005) 114(5):806–16. doi: 10.1002/ijc.20740 15609302

[3] Ferlito A Shaha A Buckley J Rinaldo A. Selective neck dissection for hypopharyngeal cancer in the clinically negative neck should it be bilateral. Acta Otolaryngol (2001) 121:329–35. doi: 10.1080/000164801300102671 11425196

[4] Affleck DG Karwande SV Bull DA Haller JR Stringham JC Davis RK. Functional outcome and survival after pharyngolaryngoesophagectomy for cancer. Am J Surg (2000) 180(6):546–50. doi: 10.1016/S0002-9610(00)00517-1 11182415

[5] Okamoto M Takahashi H Yao K Inagi K Nakayama M Nagai H. Clinical impact of using chemoradiotherapy as a primary treatment for hypopharyngeal cancer. Acta Otolaryngol Suppl (2002) 2002(547):11–4. doi: 10.1080/000164802760057491 12212584

[6] Tong DK Law S Kwong DL Wei WI Ng RW Wong KH. Current management of cervical esophageal cancer. World J Surg (2011) 35(3):600–7. doi: 10.1007/s00268-010-0876-7 21161656

[7] Putten L Bree R Doornaert PA Buter J Eerenstein SE Rietveld DH. Salvage surgery in post-chemoradiation laryngeal and hypopharyngeal carcinoma: outcome and review. Acta Otorhinolaryngol Ital (2015) 35(3):162–72. PMC4510934 26246660

[8] Liu J Zhang Y Li Z Liu S Li H Xu Z. Benefit of salvage total pharyngolaryngoesophagectomy for recurrent locally advanced head and neck cancer after radiotherapy. Radiat Oncol (2017) 12(1). doi: 10.1186/s13014-017-0900-2 PMC5658928 29073917

[9] Liu QX Qiu Y Deng XF Dai JG. Comparison of outcomes following end-to-end hand-sewn and mechanical oesophagogastric anastomosis after oesophagectomy for carcinoma: a prospective randomized controlled trial. Eur J Cardiothorac Surg (2015) 47(3):e118–123. doi: 10.1093/ejcts/ezu457 25475947

[10] Sallum RA Coimbra FJ Herman P Montagnini AL Machado MA. Modified pharyngogastrostomy by a stapler technique. Eur J Surg Oncol (2006) 32(5):540–3. doi: 10.1016/j.ejso.2006.02.022 16731315

[11] Tangoku A Hayashi H Yoshino S Ueno T Abe T Yoshimoto Y. Wire-guided transoral esophagogastrostomy for carcinoma of the cervical esophagus. J Am Coll Surg (1999) 189(3):330–3. doi: 10.1016/s1072-7515(99)00090-3 10472936

[12] Jiang M He X Wu D Han Y Zhang H Wang M. Reconstruction techniques for hypopharyngeal and cervical esophageal carcinoma. J Thorac Dis (2015) 7(3):449–54. doi: 10.3978/j.issn.2072-1439.2015.02.12 PMC4387411 25922724

[13] Rice TW Blackstone EH Rusch VW. 7th edition of the AJCC cancer staging manual: esophagus and esophagogastric junction. Ann Surg Oncol (2010) 17(7):1721–4. doi: 10.1245/s10434-010-1024-1 20369299

[14] Low DE Alderson D Cecconello I Chang AC Darling GE D'Journo XB. International consensus on standardization of data collection for complications associated with esophagectomy: Esophagectomy complications consensus group (ECCG). Ann Surg (2015) 262(2):286–94. doi: 10.1097/SLA.0000000000001098 25607756

[15] Saeki H Tsutsumi S Yukaya T Tajiri H Tsutsumi R Nishimura S. Clinicopathological features of cervical esophageal cancer: Retrospective analysis of 63 consecutive patients who underwent surgical resection. Ann Surg (2017) 265(1):130–6. doi: 10.1097/sla.0000000000001599 28009737

[16] Kuo P Sosa JA Burtness BA Husain ZA Mehra S Roman SA. Treatment trends and survival effects of chemotherapy for hypopharyngeal cancer: Analysis of the national cancer data base. Cancer (2016) 122(12):1853–60. doi: 10.1002/cncr.29962 27019213

[17] Gkika E Gauler T Eberhardt W Stahl M Stuschke M Pottgen C. Long-term results of definitive radiochemotherapy in locally advanced cancers of the cervical esophagus. Dis Esophagus (2014) 27(7):678–84. doi: 10.1111/dote.12146 24147973

[18] Grass GD Cooper SL Armeson K Garrett-Mayer E Sharma A. Cervical esophageal cancer: a population-based study. Head Neck (2015) 37(6):808–14. doi: 10.1002/hed.23678 24616217

[19] Zhang P Xi M Zhao L Qiu B Liu H Hu YH. Clinical efficacy and failure pattern in patients with cervical esophageal cancer treated with definitive chemoradiotherapy. Radiother Oncol (2015) 116(2):257–61. doi: 10.1016/j.radonc.2015.07.011 26233590

[20] Shapiro J van Lanschot JJB Hulshof MCCM van Hagen P van Berge Henegouwen MI Wijnhoven BPL. Neoadjuvant chemoradiotherapy plus surgery versus surgery alone for oesophageal or junctional cancer (CROSS): long-term results of a randomised controlled trial. Lancet Oncol (2015) 16(9):1090–8. doi: 10.1016/s1470-2045(15)00040-6 26254683

[21] Schizas D Theochari NA Ziogas IA Economopoulos KP Mylonas KS. Carcinomas of the hypopharynx and cervical esophagus: A systematic review and quality of evidence assessment. J BUON (2021) 26(1):39–50. 33721430

[22] Duan X Bai W Ma Z Yue J Shang X Jiang H. Management and outcomes of anastomotic leakage after McKeown esophagectomy: A retrospective analysis of 749 consecutive patients with esophageal cancer. Surg Oncol (2020) 34:304–9. doi: 10.1016/j.suronc.2020.06.002 32891347

